# Diagnosis and Management of Spontaneous Lumbar Venous Retroperitoneal Hematoma in Setting of Deep Venous Thrombosis: A Case Report and Algorithm

**DOI:** 10.1155/2016/3183985

**Published:** 2016-10-03

**Authors:** Joseph Tseng, Michael Leshen, Todd Chapman, Ryan Scott, Olga Kalinkin

**Affiliations:** ^1^Department of Medicine, University of California Irvine School of Medicine, 333 City Boulevard West, Suite 400, Orange, CA 92868, USA; ^2^Creighton University School of Medicine, Phoenix Regional Campus, 350 W Thomas Road, Phoenix, AZ 85013, USA; ^3^Department of Radiology, St. Joseph's Hospital, 350 W Thomas Road, Phoenix, AZ 85013, USA

## Abstract

Retroperitoneal hematoma is rare and benefits from a systematic approach to prevent morbidity and mortality. Management of such bleeds is based upon patient stability, the cause (spontaneous or posttraumatic), and source (arterial or venous). Herein, the authors describe a diagnostic and management algorithm for retroperitoneal hemorrhage with an example of a rare lumbar venous bleed under the complicated clinical setting of deep venous thrombosis.

## 1. Introduction 

Retroperitoneal hematoma is a well-known, but rare, clinical condition that can be potentially lethal. They are most frequently seen as a complication of interventions such as femoral artery catheterizations or abdominal trauma [1]. Retroperitoneal hemorrhage has a variety of etiologies that should be suspected in patients with significant groin, flank, abdominal, back pain, or hemodynamic instability following an interventional procedure. When this occurs in patients without any obvious precipitating factors, it is termed spontaneous retroperitoneal hemorrhage. Most often, these patients are anticoagulated or on hemodialysis. There is an incidence of 0.1% to 0.6% in patients receiving oral anticoagulants [2]. Despite having well-recognized clinical signs such as the Grey-Turner and Cullen signs, diagnosis of retroperitoneal hemorrhage is often delayed due to the manifestation of these signs late in the clinical course. Due to the ability of hemorrhage to rapidly exsanguinate and cause shock, the survival of the patient is dependent on rapid and accurate diagnosis.

Lumbar vein hemorrhage is rare. There have been reports of lumbar vein rupture as a complication of acute pancreatitis and a patient taking fondaparinux [3, 4]. However there has never been a report of this occurring in otherwise asymptomatic patients. In this case, bleeding was identified with CT and managed with the utilization of transcatheter venous embolization as a method of bleeding control. Here, we present a rare case of retroperitoneal hematoma formation secondary to a lumbar vein bleed in the setting of deep venous thrombosis and provide a diagnostic and management algorithm.

## 2. Case Report

A 58-year-old homeless male with a history of hypertension and prior venous thromboembolism presented to the emergency department (ED) for 1 week of weakness and fatigue. He denied any hemoptysis, dyspnea, chest pain, headache, nausea, and vomiting. Besides a heart rate of 113 bpm his physical exam was normal. His past medical history included type II diabetes. His past surgical history was significant for an infrarenal IVC filter placement 15 years ago that was still in place. He was unsure as to why it was initially placed. He had no known allergies and took atenolol, lisinopril, and metformin at home. He was not taking any oral anticoagulants. He had no family history of malignancies and bleeding disorders.

While in the ED the patient became unresponsive and ACLS was initiated and lasted for 11 minutes. The patient was intubated, resuscitated, and transferred to the intensive care unit. While in the ICU it was noted that the patient's hemoglobin dropped from 12.3 gm/dL to 6.8 gm/dL.

## 3. Differential Diagnosis

The differential diagnosis for acute anemia primarily includes blood loss and a wide variety of hemolytic processes such as microangiopathic hemolytic anemia, disseminated intravascular coagulation, sickle cell disease, and paroxysmal nocturnal hemoglobinuria just to name a few. Based on the patient's history and current clinical condition our initial concern was acute anemia secondary to blood loss and a noncontrast CT scan of the chest, abdomen, and pelvis was ordered.

## 4. Imaging Findings

**Figure 1:**
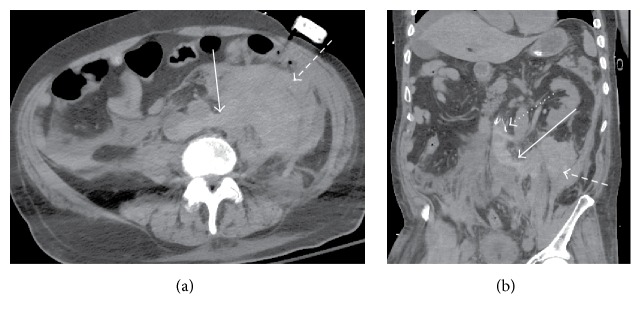
(a) Initial noncontrast CT axial image demonstrated a large high density left retroperitoneal hematoma (dashed arrow) contiguous with left site of aorta and IVC (arrow). (b) Initial noncontrast CT coronal reformatted images demonstrated a large high density left retroperitoneal hematoma (dashed arrow) contiguous with high-attenuated tubular structure arising from the left side of IVC (arrow), likely a lumbar vein. There is an expansile appearance of infrarenal IVC. IVC filter above the left inferior renal vein is also appreciated (dotted arrow).

**Figure 2:**
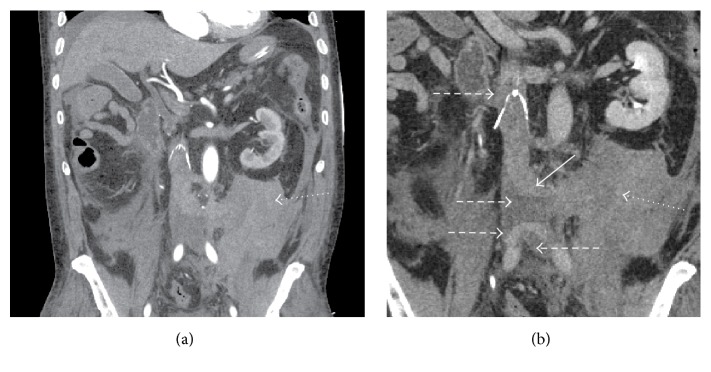
(a) Subsequent contrast CT coronal reformatted image during arterial phase demonstrated no active aortic or arterial contrast extravasation associated with a high density large retroperitoneal hematoma (dotted arrow). (b) Contrast CT coronal reformatted image during delayed (120 sec) venous phase demonstrated hyperattenuated contrast material below the IVC filter contiguous with dilated left lumbar vein (arrow) extending to left retroperitoneal hematoma (dotted arrow). Nonocclusive hypoattenuated clots (dashed arrows) are present below the IVC filter along with an irregular contour of the distal left IVC. Small nonocclusive thrombi above the IVC filter tip and in the iliac veins are also appreciated (dashed arrows).

The CT scan demonstrated a large, left-sided retroperitoneal hematoma contiguous with left site of aorta and IVC, mass effect on the bladder, and expansile appearance of IVC below the IVC filter (Figures 1(a) and 1(b)). Follow-up abdominal CT angiogram with arterial and venous phases excluded arterial etiology of retroperitoneal bleed and reveled nonocclusive IVC and bilateral common iliac vein thrombi slightly extended above the IVC filter. High attenuation left retroperitoneal hematoma was contiguous with left wall of infrarenal IVC and tubular left retroperitoneal structure of likely dilated left lumbar vein (Figures 2(a) and 2(b)). A follow-up CT angiogram of the chest was obtained due to the presence of the thrombus proximal to the patient's IVC filter and revealed multiple subsegmental pulmonary emboli in the right lower lobe of the lung.

## 5. Treatment

**Figure 3:**
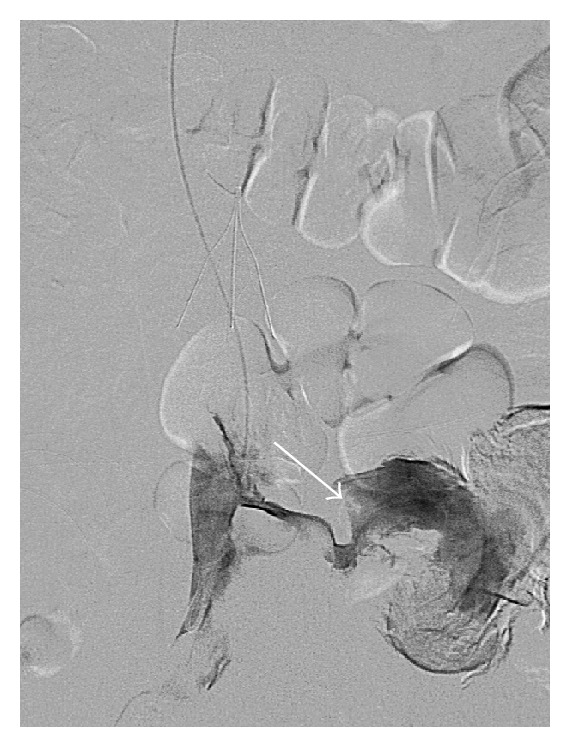
Selective catheter venogram reveals brisk active extravasation from a left lumbar vein into the left retroperitoneal hematoma (arrow).

**Figure 4:**
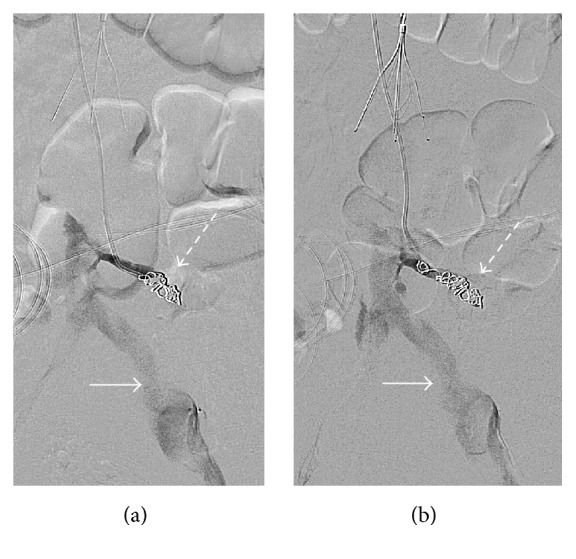
Postembolization venography after Gelfoam and coil embolization (dashed arrow) showed no residual active extravasation from this branch. A nonocclusive thrombus in the common iliac vein is noted (arrow).

The patient was referred to Interventional Radiology and an inferior venacavogram was performed. The right internal jugular vein was chosen as an access point due to operator preference and to avoid the large clot burden in the iliac veins and IVC. It was accessed with a micropuncture system, via ultrasound guidance. The micropuncture sheath was exchanged for a 5-French vascular sheath. An Omni Flush catheter was then advanced to the infrarenal IVC, distal to the patient's old IVC filter. Injection of contrast revealed brisk active extravasation from a left lumbar venous branch (Figure 3). This branch was then selectively embolized with Gelfoam slurry and multiple 0.035 embolic coils (Figures 4(a) and 4(b)). No residual extravasation was identified on postembolization venography.

**Figure 5:**
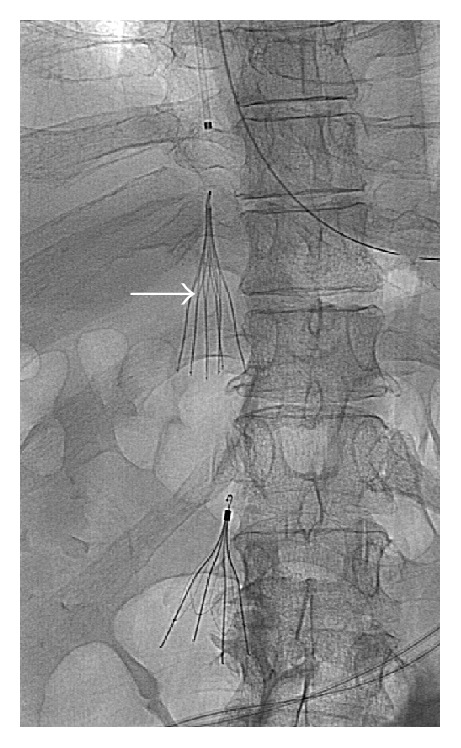
A final fluoroscopic spot view confirmed appropriate positioning of suprarenal IVC filter placement (arrow).

The IVC above the filter was then selected and an inferior venacavogram was again performed, this time showing multiple filling defects compatible with venous thrombus, including thrombus at the superior aspect of the IVC filter, outside of the filter cone. Given the acute subsegmental pulmonary emboli noted on the previous CTA chest, as well as the contraindication to oral anticoagulation, the decision was made to deploy a suprarenal IVC filter (Figure 5). The patient tolerated the procedure well with no complications and his hemoglobin stabilized to 9.3 gm/dL.

Over the next two days the patient developed persistent scrotal pain, leg swelling, and pain. This was attributed to the patients extensive clot burden. Thrombectomy and infrarenal IVC filter retrieval were then performed in an attempt to alleviate the patients swelling. Due to the large size of the clots an AngioVac cannula and circuit were used. During the procedure a large portion of clot was removed from the patient's left superficial femoral vein through the IVC. However, the infrarenal IVC was unable to be retrieved. While recovering from the procedure the patient became tachycardic, hypoxic, and hypotensive and was readmitted to the ICU. The patient's instability was most likely due to a postprocedure pulmonary embolism secondary to thrombus disruption. However, no imaging was performed to confirm this suspicion. He was then stabilized and was placed on lovenox and with the intention to bridge to warfarin for outpatient management. His hemoglobin remained stable throughout the remainder of his hospitalization and he was discharged shortly thereafter. Unfortunately the patient was then lost to follow-up and the status of either IVC filter is unknown.

## 6. Discussion

This patient presented with a complicated clinical scenario; as he had a history of thromboembolic events, yet he was actively hemorrhaging into the retroperitoneum. Furthermore, the patient had a previous infrarenal IVC filter that was involved in an occlusive clot formation. IVC filter thrombosis has been reported in 3 to 30 percent of patients following filter placement [5]. While obstruction can be asymptomatic, it can often lead to lower extremity edema, increase the risk of other sequelae of venous insufficiency, and result in the development of collateral vessels that may serve as a route for recurrent embolization [1].

### 6.1. Spontaneous Retroperitoneal Hematoma from Lumbar Vein

Retroperitoneal hematoma is a rare clinical condition, with an incidence of 0.1%, and as high as 0.6% in patients receiving oral anticoagulation therapy. It is most frequently seen as a complication of interventions such as femoral artery catheterizations and pelvic or lumbar trauma. Retroperitoneal hemorrhage has a variety of etiologies that should be suspected in patients with significant groin, flank, abdominal, back pain, or hemodynamic instability following an interventional procedure. When bleeding happens without any obvious precipitating factors, it is termed spontaneous hemorrhage. Spontaneous hemorrhage occurs most frequently in patients who are anticoagulated or on hemodialysis [6].

The pathogenesis and pathophysiology of spontaneous retroperitoneal bleeding remain unclear, but it has been hypothesized that heparin or anticoagulation-induced immune microangiopathy may play a role. In this model, unrecognized minor trauma in the microcirculation in the presence of coagulation may lead to hemorrhage. This unrecognized trauma can be due to a variety of factors, such as vomiting, coughing, or even trauma secondary to participating in sports [6].

Lumbar vein hemorrhage is rare, and lumbar vein hemorrhage leading to a retroperitoneal hematoma has previously been unreported. Lumbar veins drain blood from the abdominal wall and vertebral venous plexus into the inferior vena cava, ascending lumbar veins, renal veins, or lumbar azygous vein. While there has been a reported case of lumbar vein rupture as a complication of acute pancreatitis, there has been no report of this occurring in otherwise asymptomatic patients [3].

In our unique case we cannot completely rule out iatrogenic trauma secondary to CPR. It is possible that the chest compressions forced blood retrograde, below the filter. The thrombosed infrarenal IVC filter may have acted as a valve, slowing anterograde flow, increasing pressure in the lumbar vein, and causing it to rupture. However retroperitoneal bleeds due to CPR are extremely rare, with an incidence of 0.1% [7]. In addition to the low incidence of iatrogenic retroperitoneal hematomas secondary to CPR, the site of the lumbar vein hematoma in this patient was not near the area of chest compressions and thus makes it less likely to be the cause of the bleed.

### 6.2. Radiological Evaluation of Retroperitoneal Hematoma

**Figure 6:**
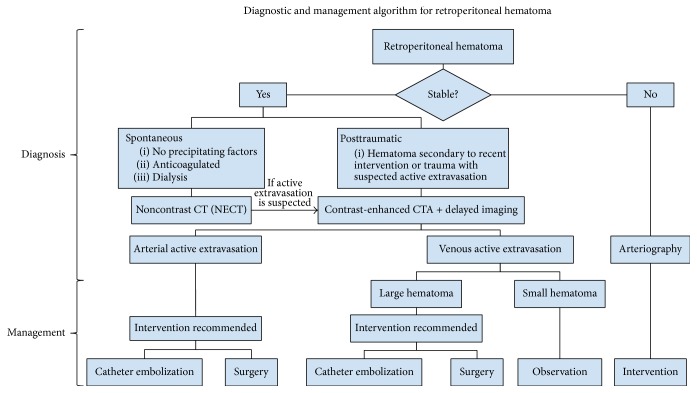
Diagnostic algorithm for evaluation of a retroperitoneal hematoma.

The initial assessment of a patient with a suspected retroperitoneal hematoma requires evaluation for shock. Patients should forgo CT/CTA and go directly to angiography if there is high suspicion of a retroperitoneal hematoma and the patient has abnormal vital signs such as hypotension, tachycardia, decreased urine output, or altered mental status. If a patient is stable, then a more methodical radiological approach may be utilized (Figure 6). In our case, after noting a severe drop in the patient's hemoglobin a CT abdomen was ordered. Noncontrast CT revealed left retroperitoneal hematoma contiguous with the aorta, IVC, and retroaortic sentinel clot.

Identification of the etiology of bleeding is the next critical step for selection of intervention, whether it may be surgical, interventional radiological, or medical. Firstly, it must be determined whether the bleed is of arterial or venous origin. If the bleed is of arterial origin, the next step is to determine whether it is from the aorta or aortic branch. If the bleed is of venous origin, the next step is to determine whether it is from the inferior vena cava or other small veins.

A subsequent CT angiogram was performed, which excluded an aortic or arterial origin of bleeding. The large left retroperitoneal hematoma was contiguous with the IVC/lumbar vein and was highlighted by the sentinel clot. IVC thrombi below and above the IVC filter as well as the segmental pulmonary emboli in spite of the presence of the IVC filter were identified.

Arterial vascular bleeding requires immediate vascular intervention with coil embolization. A venous bleed with a large hematoma and slow contrast extravasation requires intervention. On the other hand, slow venous bleeding from small vessels can be managed with observation. The decision was then made to proceed with a catheter venous angiogram to identify the site of the venous bleed and to provide further management with insertion of an IVC filter. In the case of our patient the IVC filter was placed above the renal veins. It was previously thought that suprarenal IVC filters would increase the risk of renal vein thrombosis due to thrombus accumulation. However, several studies have shown there no increased risk associated with suprarenal IVC filters versus infrarenal IVC filters [8, 9]. The most common complication of suprarenal IVC filters is filter migration due to its variability of blood volume and venous return [10].

## Additional Points

*Learning Points*. (1) Spontaneous retroperitoneal hematomas are a rare event that typically occurs in patients that are receiving anticoagulation or hemodialysis. (2) Retroperitoneal hematomas may benefit from an algorithm-based approach to management. (3) Management of pulmonary embolism or DVT can be complicated by active bleeding.
